# The Effects of Indirect and Direct Modulation of Endocannabinoid System Function on Anxiety-Related Behavior in Mice Assessed in the Elevated Plus Maze Test

**DOI:** 10.3390/molecules30040867

**Published:** 2025-02-13

**Authors:** Marta Kruk-Slomka, Agnieszka Dzik, Grazyna Biala

**Affiliations:** 1 Department of Pharmacology and Pharmacodynamics, Medical University of Lublin, 4a Chodzki Str., 20-093 Lublin, Poland; agnieszkadzik2016@gmail.com; 2 Experimental Medicine Center (OMD), Medical University of Lublin, Jaczewskiego 8D, 20-090 Lublin, Poland

**Keywords:** anxiety, endocannabinoid system, cannabinoid receptor ligands, mice, elevated plus maze test

## Abstract

Background: The endocannabinoid system (ECS) is one of the most important systems modulating functions in the body. The ECS, via cannabinoid (CB: CB1 and CB2) receptors, endocannabinoids occurring in the brain (e.g., anandamide (AEA) and 2-arachidonoylglycerol (2-AG)) and enzymes degrading endocannabinoids in the brain (fatty-acid amide hydrolase (FAAH) and monoacylglycerol lipase (MAGL)), plays a key role in the regulation of mood and anxiety. However, the effects of cannabinoid compounds on anxiety-related responses are complex and yield mixed results depending on the type of pharmacological manipulation (direct or indirect) of functions of the ECS, as well as the kinds of cannabinoids, dosage and procedure. Methods: The aim of this study was to determine and compare the influence of the direct (via CB receptors ligands) and indirect (via inhibition of enzymes degrading endocannabinoids in the brain) pharmacological modulation of ECS function on anxiety-like responses in mice in the elevated plus maze (EPM) test. For this purpose, in the first step of the experiments, we used selected ligands of CB1, CB1/CB2 and CB2 receptors to assess which types of CB receptors are involved in anxiety-related responses in mice. Next, we used inhibitors of FAAH (which breaks down AEA) or MAGL (which breaks down 2-AG) to assess which endocannabinoid is more responsible for anxiety-related behavior in mice. Results: The results of our presented research showed that an acute administration of CB1 receptor agonist oleamide (5–20 mg/kg) had no influence on anxiety-related responses and CB1 receptor antagonist AM 251 (0.25–3 mg/kg) had anxiogenic effects in the EPM test in mice. In turn, an acute administration of mixed CB1/CB2 receptor agonist WIN55,212-2 used at a dose of 1 mg/kg had an anxiolytic effect observed in mice in the EPM test. What is of interest is that both the acute administration of a CB2 receptor agonist (JWH 133 at the doses of 1 and 2 mg/kg) and antagonist (AM 630 at the doses of 0.5–2 mg/kg) had anxiogenic effects in this procedure. Moreover, we revealed that an acute administration of only FAAH inhibitor URB 597 (0.3 mg/kg) had an anxiolytic effect, while MAGL inhibitor JZL 184 (at any used doses (2–40 mg/kg)) after an acute injection had no influence on anxiety behavior in mice, as observed in the EPM test. Conclusions: In our experiments, we confirmed the clearly significant involvement of the ECS in anxiety-related responses. In particular, the pharmacological indirect manipulation of ECS functions is able to elicit promising anxiolytic effects. Therefore, the ECS could be a potential target for novel anxiolytic drugs; however, further studies are needed.

## 1. Introduction

The endocannabinoid system (ECS) is a complex cell-signaling system that plays a key role in regulating many physiological and psychological/central functions, including memory and learning, sleep, appetite, pain sensation and immune function, as well as mood and responses to stress and anxiety [1,2]. Such extensive involvement of the ECS in many processes and body homeostasis is related to the complex components that are part of the ECS. The ECS consists of three primary components, including cannabinoid (CB) receptors, endocannabinoids that occur naturally in the brain and enzymes that break down endocannabinoids in the brain. Endocannabinoids are small functional molecules that have been isolated from arachidonic acid, a membrane layer. The two most-known main endocannabinoids in the body are anandamide (AEA) and 2-arachidonoylglycerol (2-AG), and they are directly linked with a class of G-protein coupled receptors. AEA plays a role in regulating mood and emotions and has a general modulatory effect on brain reward circuits. Numerous reports also suggest that AEA plays a role in the addictive effects of other drugs and may serve as a behavioral reinforcer in animal addiction models. Moreover, AEA can act as a behavior enhancer under certain conditions, while, in other cases, it may elicit aversive behaviors or increase anxiety level [3,4]. 2-AG is more abundant and is involved in a wide range of functions, including immune response and inflammation regulation. These endocannabinoids in the brain are broken down by specific enzymes: fatty acid amide hydrolase (FAAH) breaks down AEA and monoacylglycerol lipase (MAGL) breaks down 2-AG [3]. Endocannabinoids in the brain are similar in structure to the active plant compounds occurring in the Cannabis sativa. The most well-known phytocannabinoids are tetrahydrocannabinol (THC) and cannabidiol (CBD) [5].

Endocannabinoids and both plant and synthetic cannabinoid compounds act on the body through specific cannabinoid (CB) receptors of type 1 (CB1) and type 2 (CB2). CB1 receptors are located mainly in the central nervous system (CNS). These receptors are widely distributed across various brain structures, with the highest concentration found in regions associated with cognitive functions, movement or emotions, such as the amygdala, hippocampus, septum (septum pellucidum), cerebral cortex, globus pallidus, substantia nigra, cerebellum and caudate nucleus. CB1 receptors are mainly present in structures of the brain’s limbic system, as well as in lower densities in various peripheral tissues, both in the fibers of sensory nerves and in the autonomic nervous system. CB1 receptors are located presynaptically on glutamatergic and gamma-aminobutyric acid (GABA)-ergic nerve fiber terminals. In the hippocampus, a brain structure crucial for memory processes, CB1 receptors are primarily found on inhibitory GABAergic interneurons. Due to their specific location, CB1 receptors are able to control both cognitive functions and behaviors related to emotions such as stress, fear and anxiety. CB2 receptors are located primarily in the peripheral tissues and immune cells. They play a role in regulating inflammation and immune responses. However, the CB2 receptor has been identified also in the CNS, mainly in the microglia, and CB2 receptor expression can be upregulated in microglia during inflammatory states. Indeed, CB2 receptors modulate both excitatory and inhibitory synaptic transmission in the hippocampus. It has also been shown that CB2 receptor activation leads to pain reduction and impulsive behaviors. The literature data suggest that CB2 receptors play a role in a variety of psychiatric disorders. Polymorphism of the gene encoding the CB2 receptor is closely associated with schizophrenia, depression, anxiety and bipolar disorders [6,7,8,9,10].

Thus, cannabis compounds, due to a multidirectional mechanism of action, are associated with regulating a variety of processes [11,12,13]. Among others, the ECS plays a crucial role in regulating processes related to anxiety; therefore, understanding the role of the ECS in anxiety is the subject of many pre- and clinical studies. On the one hand, modulating the functions of the ECS may have a beneficial effect on anxiety, but there is also considerable evidence that links use of cannabis with increased anxiety [1,13,14,15,16,17,18]. Research connecting the effects of compounds acting by modulation of the ECS with anxiety is complex and yields mixed and contradictory results, depending on the type of cannabinoid, compound and dosage and the specific anxiety-related behaviors being studied. It has been noted that CB receptor ligands can affect anxiety-like behaviors in a variety of ways—both anxiolytic (anxiety-reducing) and anxiogenic (anxiety-increasing) effects have been revealed. Biphasic effects of cannabinoids in the context of anxiety assessment in mice have been widely documented, suggesting that cannabinoids may exhibit both anxiolytic and anxiogenic effects depending on factors such as dose, timing and specific receptor activation. There are scientific reports suggesting that the effects of CB1 receptor agonists are dose-dependent, i.e., low doses have anxiolytic effects while high doses have anxiety-producing effects. For example, low doses of phytocannabinoids, such as THC, often induce anxiolytic effects, while higher doses can lead to anxiogenic responses. This biphasic pattern is thought to result from differential activation of CB receptors and probably as a consequence of cannabinoid regulation of GABA/glutamate balance [13,15,18]. The anxiolytic and anxiogenic potential of phytocannabinoids has also been documented in humans. Epidemiological studies have typically suggested an anxiolytic effect from consuming CBD or THC, as well as whole Cannabis sativa plants, whereas clinical studies in humans have commonly reported an anxiogenic response to THC, particularly at higher doses [19].

What is of interest is that scientists have conducted a number of experiments to define the role of compounds directly acting on the function of the ECS using CB receptor ligands and there is little information associated with the indirect influence of the modulation of ECS activity on emotions, including anxiety. What is more, a direct modulation of the ECS through CB receptor ligands may not be effective in treating emotional disorders and, as we mentioned, is often associated with adverse effects. An alternative and promising approach to avoid these side effects focuses on indirect activation of the ECS via inhibition of endocannabinoid-degrading enzymes in the brain (using FAAH or/and MAGL inhibitors). Some studies suggest that inhibiting FAAH and/or MAGL could enhance endocannabinoid signaling, leading to increased levels of AEA or/and 2-AG, which may represent potential targets for developing new pharmacotherapeutics for anxiety-related disorders. It is worth noting, however, that there is scientific evidence also indicating typical biphasic effects of endocannabinoids, e.g., AEA, as described above [20,21,22]. Following that, the results of these studies remain unclear and do not answer for the second main question of how indirect manipulation of this system can affect anxiety-related behavior.

Based on the cited scientific reports, both on clinical trials as well as animal experiments, we obtain unclear and contradictory results concerning the role of the ECS in anxiety-related behavior. Therefore, our work attempts to assess and compare the influence of a variety of compounds modulating the ECS function directly and indirectly on anxiety-related behaviors in mice. In our experiments, we used both a CB1 receptor agonist (oleamide) and antagonist (AM 251), as well as a CB1/CB2 receptor mixed agonist (WIN 55,212) and CB2 receptor agonist (JWH 133) and antagonist (AM 630), to evaluate the direct influence of ECS on the anxiety-related action in mice. In the next step, we used inhibitors of enzymes that break down endocannabinoids in the brain (FAAH inhibitor URB 597 and MAGL inhibitor JZL 184) to evaluate the indirect impact of the ECS on the anxiety behavior in mice. We used the EPM test, which has been validated for the evaluation of anxiety in mice. The results obtained from our studies will expand knowledge about the involvement of the ECS in anxiety-provoking behavior and provide further information on the relationship between cannabis use and anxiety. Investigating and understanding the involvement of the ECS in these processes could lead to the discovery of novel promising strategies for the development of more precise and safer anxiolytic therapies using pharmacological direct or/and indirect modulation of the function of the ECS.

## 2. Results

### 2.1. The Influence of an Acute Administration of Compounds Acting Directly on the ECS on the Anxiety-Related Responses in Mice in the EPM Test

#### 2.1.1. CB1 Receptor Ligands: Oleamide and AM-251

**Figure 1:**
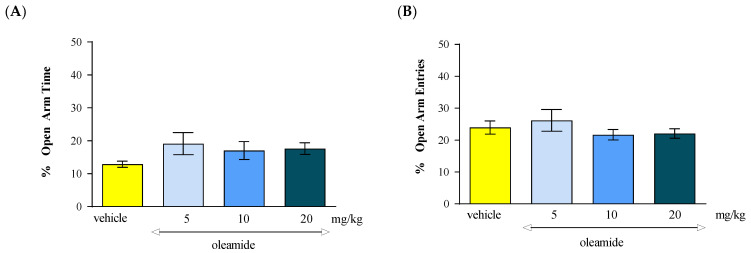
The effect of an acute administration of the CB1 receptor agonist oleamide on the anxiety-related behavior in the EPM test in mice. Mean (+/− SEM) percentage of time spent in open arms (**A**) and percentage of open arm entries (**B**) in the EPM, 30 min after an acute ip injection of oleamide (5–20 mg/kg) or vehicle in mice; *n* = 8.

Statistical analysis of the results using the one-way ANOVA showed that an acute administration of CB1 receptor agonist oleamide at doses of 5 mg/kg, 10 mg/kg and 20 mg/kg caused no statistically significant differences between the groups of animals receiving oleamide in the above-mentioned doses and the control group (vehicle), both in relation to the percentage of time spent by the animals in the open arms of the EPM [F(3.31) = 1.264; *p* = 0.3058] (Figure 1A) and the percentage of the number of entries into these arms [F(3.31) = 0.8256; *p* = 0.4909] (Figure 1B).

Number of entries into closed arms of the EPM: The locomotor activity of mice expressed by the number of entries into closed arms in the above-mentioned experimental groups did not change significantly (one-way ANOVA, F(3.31) = 0.2685; *p* = 0.8475) (Table 1).

**Figure 2:**
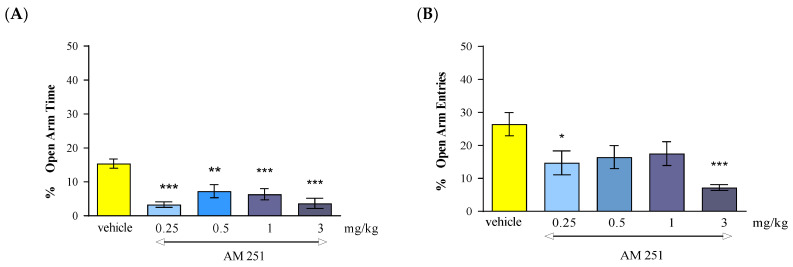
The effect of an acute administration of the CB1 receptor antagonist AM 251 on anxiety-related behavior in the EPM test in mice. Mean (+/− SEM) percentage of time spent in open arms (**A**) and percentage of open arm entries (**B**) in the EPM, 30 min after an acute ip injection of AM 251 (0.25–3 mg/kg) or vehicle in mice; *n* = 8. * *p* < 0.05; ** *p* < 0.01; *** *p* < 0.001 vs. vehicle control group, Tukey’s test.

Statistical analysis of the results using the one-way ANOVA method showed that a single administration of CB1 receptor antagonist AM 251 at doses of 0.25 mg/kg, 0.5 mg/kg, 1 mg/kg and 3 mg/kg caused statistically significant differences between the groups of animals receiving AM 251 in the above-mentioned doses and the control group (vehicle), both in relation to the percentage of time spent by the animals in the open arms of the EPM [F(4.39) = 10.68; *p* < 0.0001] and the percentage of the number of entries into these arms [F(4.39) = 4.595; *p* = 0.0044]. Tukey’s post hoc test confirmed that AM 251 administered at a range of doses from 0.25 to 3 mg/kg reduced the percentage of time spent by mice in the open EPM arms (*p* < 0.01 for the dose of 0.5 mg/kg and *p* < 0.001 for the doses of 0.25, 1 and 3 mg/kg) compared to the control group (Figure 2A) and the percentage of the number of entries into the open arms of EPM (*p* < 0.05 for the dose of 0.25 mg/kg and *p* < 0.001 for the dose of 3 mg/kg) in individual groups receiving AM 251 in relation to the control group (Figure 2B).

Number of entries into EPM closed arms: The locomotor activity of mice expressed by the number of entries into closed arms in the above-mentioned experimental groups did not change significantly (one-way ANOVA, F(4.39) = 0.4662; *p* = 0.7601) (Table 2).

#### 2.1.2. CB1/CB2 Receptor Ligands: WIN 55,212-2

**Figure 3:**
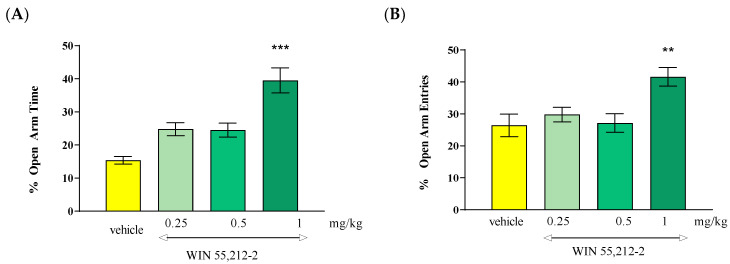
The effect of an acute administration of the mixed CB1/CB2 receptor agonist WIN 55,212-2 on anxiety-related behavior in the EPM test in mice. Mean (+/− SEM) percentage of time spent in open arms (**A**) and percentage of open arm entries (**B**) in the EPM, 30 min after an acute ip injection of WIN 55,212-2 (0.25–1 mg/kg) or vehicle in mice; *n* = 8. ** *p* < 0.01 and *** *p* < 0.001 vs. vehicle control group, Tukey’s test.

Statistical analysis of the results using the one-way ANOVA method showed that an acute administration of mixed CB1/CB2 receptor agonist WIN 55,212-2 at doses of 0.25 mg/kg, 0.5 mg/kg and 1 mg/kg caused statistically significant differences between the groups of animals receiving WIN 55,212-2 administered at the above-mentioned doses and the control group (vehicle), both in relation to the percentage of time spent by the animals in the open arms of the EPM [F(3.31) = 16.75; *p* < 0.0001] and the percentage of the number of entries into these arms [F(3.31) = 5.741; *p* = 0.0034]. Tukey’s post hoc test confirmed that an acute injection of WIN 55,212-2 administered at a dose of 1 mg/kg caused a statistically significant increase in the percentage of time spent in the open EPM arms (*p* < 0.001) (Figure 3A). Moreover, an acute injection of WIN 55,212-2 administered at a dose of 1 mg/kg significantly increased the percentage of the number of entries into the open arms of the EPM (*p* < 0.01) (Figure 3B). These values were compared to the control group receiving the vehicle.

Number of entries into EPM closed arms: The locomotor activity of mice expressed by the number of entries into closed arms in the above-mentioned experimental groups did not change significantly (one-way ANOVA, F(3.31) = 1.911; *p* = 0.1508) (Table 3).

#### 2.1.3. CB2 Receptor Ligands: JWH 133 and AM 630

**Figure 4:**
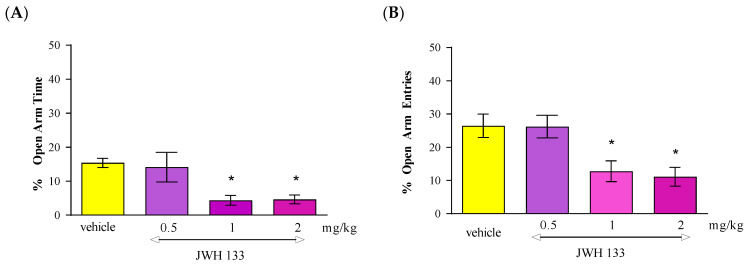
The effect of an acute administration of the CB2 receptor agonist JWH 133 on anxiety-related behavior in the EPM test in mice. Mean (+/− SEM) percentage of time spent in open arms (**A**) and percentage of open arm entries (**B**) in the EPM, 30 min after an acute ip injection of JWH 133 (0.5–2 mg/kg) or vehicle in mice; *n* = 8–10. * *p* < 0.05 vs. vehicle control group, Tukey’s test.

Statistical analysis of the results using the one-way ANOVA method showed that an acute administration of CB2 receptor agonist JWH 133 at doses of 0.5 mg/kg, 1 mg/kg and 2 mg/kg caused statistically significant differences between groups of animals receiving JWH 133 in the above-mentioned doses and the control group (vehicle), both in relation to the percentage of time spent by the animals in the open arms of the EPM [F(3.35) = 6.611; *p* = 0.0013] as well as the percentage of the number of entries into these arms [F(3.35) = 6.685; *p* = 0.0012]. Tukey’s post hoc test showed that an acute injection of JWH 133 administered at doses of 1 mg/kg and 2 mg/kg resulted in a statistically significant reduction in the percentage of time spent in open EPM arms (*p* < 0.05) (Figure 4A) and reduced the percentage of the number of entries into the open arms of the EPM (*p* < 0.05) (Figure 4B). These values were compared to the control group receiving the vehicle.

Number of entries into EPM closed arms: The locomotor activity of mice expressed by the number of entries into closed arms in the above-mentioned experimental groups did not change significantly (one-way ANOVA, F(3.35) = 0.5579; *p* = 0.6467) (Table 4).

**Figure 5:**
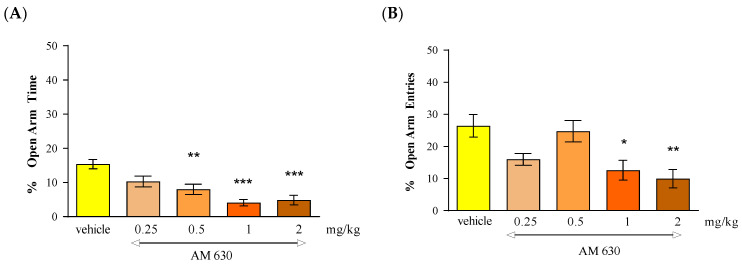
The effect of an acute administration of the CB2 receptor antagonist AM on anxiety-related behavior in the EPM test in mice. Mean (+/− SEM) percentage of time spent in open arms (**A**) and percentage of open arm entries (**B**) in the EPM, 30 min after an acute ip injection of AM 630 (0.25–2 mg/kg) or vehicle in mice; *n* = 8–10. * *p* < 0.05, ** *p* < 0.01 and *** *p* < 0.01 vs. vehicle control group, Tukey’s test.

Statistical analysis of the results using the one-way ANOVA method showed that an acute administration of CB2 receptor antagonist AM 630 at doses of 0.25 mg/kg, 0.5 mg/kg, 1 mg/kg and 2 mg/kg caused statistically significant differences between the groups of animals receiving AM 630 in the above-mentioned doses and the control group, both in relation to the percentage of time spent by the animals in the open arms of the EPM [F(4.44) = 10.68; *p* < 0.0001] and the number of entries into these arms [F(4.44) = 5.902; *p* = 0.0008]. Tukey’s post hoc test showed that an acute injection of AM 630 administered at doses of 0.5 mg/kg, 1 mg/kg and 2 mg/kg caused a statistically significant reduction in the percentage of time spent in the open EPM arms (*p* < 0.01 for dose 0.5 mg/kg and *p* < 0.001 for doses of 1 mg/kg and 2 mg/kg)) (Figure 5A). Moreover, an acute injection of AM 630 administered at doses of 1 mg/kg and 2 mg/kg significantly reduced the percentage of number of entries into the open arms of the EPM (*p* < 0.05 for a dose of 1 mg/kg and *p* < 0.01 for a dose of 2 mg/kg) (Figure 5B). These values were compared to the control group receiving the vehicle.

Number of entries into EPM closed arms: The locomotor activity of mice expressed by the number of entries into closed arms in the above-mentioned experimental groups did not change significantly (one-way ANOVA, F(4.44) = 0.2943; *p* = 0.8799) (Table 5).

### 2.2. The Influence of an Acute Administration of Compounds Acting Indirectly on the ECS on Anxiety-Related Responses in Mice in the EPM Test

#### FAAH or MAGL Inhibitors: URB 597 and JZL 194

**Figure 6:**
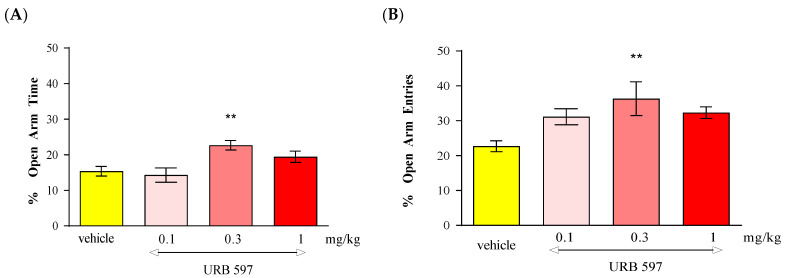
The effect of an acute administration of the FAAH inhibitor URB 597 on anxiety-related behavior in the EPM test in mice. Mean (+/− SEM) percentage of time spent in open arms (**A**) and percentage of open arm entries (**B**) in the EPM, 30 min after an acute ip injection of URB 597 (0.1–1 mg/kg) or vehicle in mice; *n* = 8; ** *p* < 0.01 vs. vehicle control group, Tukey’s test.

Statistical analysis of the results using the one-way ANOVA method showed that an acute administration of FAAH inhibitor URB 597 at doses of 0.1 mg/kg, 0.3 mg/kg and 1 mg/kg caused statistically significant differences between the groups of animals receiving URB 597 in the above-mentioned doses and the control group, both in relation to the percentage of time spent by the animals in the open arms of the EPM [F(3.31) = 5.804; *p* = 0.032] and the percentage of the number of entries into these arms [F(3.31) = 3.860; *p* = 0.0198]. Tukey’s post hoc test confirmed that an acute injection of URB 597 administered at a dose of 0.3 mg/kg caused a statistically significant increase in the percentage of time spent in the open EPM arms (*p* < 0.01) (Figure 6A) and in the percentage of the number of entries into the open arms of the EPM (*p* < 0.01) (Figure 6B). These values were compared to the control group receiving the vehicle.

Number of entries into EPM closed arms: The locomotor activity of mice expressed by the number of entries into closed arms in the above-mentioned experimental groups did not change statistically significantly (one-way ANOVA, F(3.31) = 0.1465; *p* = 0.9311) (Table 6).

**Figure 7:**
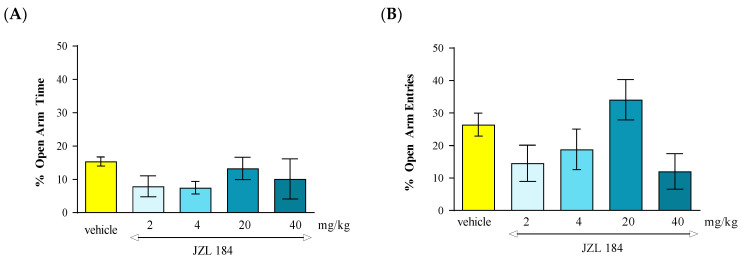
The effect of an acute administration of the FAAH inhibitor JZL 194 on anxiety-related behavior in the EPM test in mice. Mean (+/− SEM) percentage of time spent in open arms (**A**) and percentage of open arm entries (**B**) in the EPM, 30 min after an acute ip injection of JZL 194 (2–40 mg/kg) or vehicle in mice; *n* = 8.

Statistical analysis of the results using the one-way ANOVA method showed that an acute administration of MAGL inhibitor JZL 184 at doses of 2 mg/kg, 4 mg/kg, 20 mg/kg and 40 mg/kg did not cause statistically significant differences between groups of animals receiving JZL 184 in the above-mentioned doses and the control group, both in relation to the percentage of time spent by the animals in the open arms of the EPM [F(4.39) = 0.9298; *p* = 0.4579] as well as the percentage of number of entries into these arms [F(4.39) = 2.716; *p* = 0.0454]. Tukey’s post hoc test confirmed that an acute injection of JZL 184 administered at doses of 2 mg/kg, 4 mg/kg, 20 mg/kg and 40 mg/kg had no statistically significant effect on the percentage of time spent in the open arms. EPM and the percentage of number of entries into the open arms of the EPM compared to the control group receiving vehicle (Figure 7A and 7B, respectively).

Number of entries into EPM closed arms: The locomotor activity of mice expressed by the number of entries into closed arms in the above-mentioned experimental groups did not change statistically significantly (one-way ANOVA, F(4.39) = 0.2572; *p* = 0.9033) (Table 7).

## 3. Discussion

In the first step of our experiments, we evaluated the indirect influence of the ECS on anxiety-like effects in mice using the EPM test. For this purpose, we used selected ligands of CB1, CB1/CB2 and CB2 receptors to assess which type of CB receptor is mainly involved in anxiety-related responses in mice.

The results of our presented research showed that the CB1 receptor agonist oleamide administered at doses of 5, 10 and 20 mg/kg had no effect on anxiety-related responses in mice observed in the EPM test. In turn, the CB1 receptor antagonist AM 251 administered at doses of 0.25, 0.5, 1 and 3 mg/kg had an anxiogenic effect. Moreover, the acute injection of mixed CB1/CB2 receptor agonist WIN55,212-2 at a dose of 1 mg/kg had an anxiolytic influence evaluated in the EPM test in mice.

In the case of CB2 receptor involvement in anxiety-related behavior in mice, we revealed that an acute administration of CB2 receptor agonist JWH 133 and also the CB2 receptor antagonist AM 630 had a similar influence on the parameters measured in the EPM test. It was shown that the CB2 receptor agonist JWH 133 injected at doses of 1 and 2 mg/kg exhibited anxiogenic effects, and an identical effect was observed after an acute injection of the CB2 receptor antagonist AM 630 injected at doses of 0.5, 1 and 2 mg/kg.

Our results should be discussed in the context of other available data that indicate that cannabinoids compounds can significantly modulate various behaviors and emotions, both in terms of mood and anxiety in animals and humans [23]. However, these reports are not unequivocal. According to some scientists, CB1 receptor agonists exhibit anxiogenic or anxiolytic effects in rodents, depending on the administered dose—low doses induce anxiolytic effects while higher doses induce anxiogenic effects [13,15,22]. It has also been reported that intracerebral administration of CB1 receptor agonists causes anxiolytic and antidepressant effects [23,24,25,26]. For example, CB1 receptor agonist THC shows anxiolytic effects in humans; thus, this compound can also be anxiogenic in rodents, depending on the dose and context [27]. Low doses of THC can reduce anxiety-like behavior in the EPM test, where rodents are tested for anxiety based on their reluctance to enter open spaces. Higher doses of THC are often associated with increased anxiety or paranoia [28,29,30]. What is of interest is that, in experimental animal models, THC, administered at low doses, mimics the anxiolytic effects of diazepam [31,32]. Moreover, in addition to their rewarding effects, cannabinoids can also induce aversive effects, especially anxiety and panic attacks, especially after very high doses of the drugs [32,33,34]. It has also been shown that they can induce conditioned aversion, especially when animals are placed in an environment that they associate with previous drug administration [35]. In turn, the administration of CBD, a non-psychoactive cannabinoid, has gained significant attention for its potential anxiolytic properties. Unlike THC, CBD is generally considered to reduce symptoms of anxiety, and its effects have been widely explored in both human and animal studies. For example, CBD has been found to reduce anxiety in commonly used models for assessing anxiety in rodents, e.g., the light/dark box (LDB) test, the EPM test, and the open field test (OFT) [36,37,38,39]. However, there is also a contradiction in behavioral outcomes of CBD treatment, with some studies reporting anxiogenic-like effects in rodents [40,41].

In the context of our results, interesting data described the influence of another CB1 receptor agonist—oleamide—in various physical and mental functions in the body. The impact of that compound on the CNS is especially of interest, especially concerning memory and learning, sleep, mood and anxiety [42,43]. Some studies have suggested that oleamide may have positive effects on cognition and depressive-related behavior; as such, this cannabinoid compound shows a calming impact and may decrease the anxiety level in animal models [44]. Unfortunately, in our studies, oleamide at the doses used (5–20 mg/kg) did not affect the level of anxiety in mice in the EPM test; however, a tendency toward increasing in specific parameters of the EPM (the percentage of the time spent in and the number of entries into the open arms of the EPM) was maintained, which may probably indicate an anxiolytic potential of this compound. However, this direction of action was not confirmed in our study due to the lack of statistical significance.

CB1 receptor activation (due to their localization in the CNS and the increase in endocannabinoids level in the brain) is often associated with reduced anxiety. As a result of this localization (mainly in the brain structures involved in emotional control, including basolateral amygdala, cortical regions and the hippocampus), CB1 activation might have a complex pattern of influence on neurotransmitters known to modulate anxiety. Thus, the anxiolytic effects of oleamide are probably connected with the interaction with CB receptors (mainly CB1) and interaction with specific various neurotransmitters and systems. Oleamide interacts with GABAergic and serotoninergic systems, enhancing these signaling pathways. Following that, these stimulations lead to an anxiolytic effect that may be similar to effects of other CB1 receptor agonists described above [42,43,44].

Therefore, CB1 receptor antagonists may potentially have the opposite effect, increasing anxiety-related responses in animal models. Many scientific publications report that single injections of CB1 receptor antagonists rimonabant and AM 251 cause an increase in aversive behaviors associated with generalized anxiety disorders [17,45,46,47,48]. Rimonabant was used successfully in the treatment of obesity, but it also showed a number of side effects, such as anxiogenic effects, decreased mood and induced pro-suicidal behaviors. There are also scientific studies indicating the lack of effect of rimonabant on anxiety-like behaviors assessed in the EPM test in mice within the range of the tested doses [23,49].

In the case of AM 251, an analogue of rimonabant that is a potent and selective CB1 receptor antagonist but that, unlike rimonabant, has no activity at the novel receptor, the literature data have shown that this compound also has an anxiogenic-like profile. In maze-naïve mice, the lower dose of AM-251 (1.5 mg/kg) significantly reduced the percentage of open arm time and increased grooming while the higher dose (3.0 mg/kg) additionally reduced the percentage of open arm entries and total head dipping, and increased closed-arm returns in the EPM test [50,51]. These results are in accordance with the results obtained from our study. We also revealed that AM 251 tends to increase anxiety-like behaviors in mice in the EPM test. However, there are also scientific reports indicating the anxiogenic effect of AM 251, observed in wild-type mice, as well as the lack of any effect on mice lacking the gene encoding the CB1 receptor [52]. Moreover, AM 251 has the potential to reverse the sedative effect induced by diazepam in rats in the EPM test [53].

In turn, another CB1 receptor antagonist, AM 281, did not influence anxiety reaction in the dark/light box (LDB) test when given alone [54]. Similarly, another CB1 receptor antagonist, AM 4113, has no effect on anxiety reactions. It has also been shown that AM 4113, to a much lesser extent than AM 251, affects the activation of neurons in brain structures key to anxiety reactions, i.e., the amygdala [55]. It is hypothesized that this effect is related to the fact that AM 251 also exhibits activity as a reversible agonist of CB1 receptors, whereas AM 4113 only exhibits antagonist effects at this receptor.

To clearly assess the role of CB receptors in anxiety-related processes, in subsequent experiments, the next compound that we tested was the mixed CB1/CB2 receptor agonist WIN 55,212-2. We revealed that the administration of this cannabinoid compound at a dose of 1 mg/kg produced an anxiolytic effect, assessed in the EPM test in mice. Lower doses do not affect anxiety-like behaviors. These results are only partially consistent with the results of other researchers because, in some animal studies, WIN 55,212-2 might cause anxiety or other negative psychological effects while other studies suggest potential anxiolytic effects, depending on the dosage, context, individual differences and circumstances of experience. The researchers described that a dose of 0.25 mg/kg caused an anxiolytic effect while a high dose of 1.25 mg/kg showed an anxiogenic effect [56,57]. After the higher dose, WIN 55,212-2 (5 mg/kg) induced an anxiogenic-like effect accompanied by motor inhibition in the LDB test [54]. These results are consistent with the effects of experiments conducted using other mixed CB receptor agonists: HU-210 and CP 55940. These cannabinoid compounds, when administered at high doses, also intensify anxiety reactions in mice [17,25,26,45,46]. What is of interest is that both effects of WIN 55, 212-2, i.e., the anxiogenic-like and the sedative one, were attenuated by a CB1 receptor antagonist, AM 281, suggesting mainly the involvement of CB1 receptors [54].

More complicated and unclear is the role of CB2 receptors in emotions because the CB2 receptor is mostly expressed in peripheral tissues, including the immune system. Most evidence suggests that cannabinoid ligands acting on CB2 receptors could have less direct impact on mood and behavior compared to those targeting CB1 receptors, but they may modulate anxiety-related responses through immune system effects, e.g., modulating inflammation and potentially affecting neuroinflammation, which may contribute to anxiety disorders [5,6,7]. We have found some scientific reports that describe the influence of CB2 receptor ligands on emotions, mood and anxiety reactions. However, these data are so often contradictory. The available literature data results have shown their antidepressant, anxiolytic or anxiogenic effects, or they show no effect on anxiety reactions at all [58,59]. The diversity of the results obtained may be due to many factors, including the strain of animals used in the experiments, the experimental conditions, the type of procedure used to assess the results [60], the drugs previously used, differences in the doses used and the initial level of anxiety [61].

In our experiments, we revealed that both an acute administration of a CB2 receptor agonist (JWH 133 at the doses of 1 and 2 mg/kg) and antagonist (AM 630 at the doses of 0.5–2 mg/kg) had anxiogenic effects in the EPM test in mice. These results are partially consistent with the results available in the scientific literature. In some studies, CB2 receptor ligands may be used to explore their potential anxiolytic or anxiogenic effects through animal models. Acute administration of JWH 133 (0.5–2 mg/kg, ip) failed to produce any effect observed in two animal anxiety models (LDB and EPM). In turn, acute administration of AM 630 (1–3 mg/kg, ip) increased anxiety and, additionally, this effect of AM 630 (3 mg/kg) was blocked by pre-treatment with JWH 133 (2 mg/kg). Chronic administration of JWH 133 (0.5–2 mg/kg, ip, twice a day) for a treatment total of 7 days increased anxiety-like behavior whereas chronic AM 630 treatment (1–3 mg/kg, ip, twice a day) produced a significant anxiolytic effect observed in LDB and EPM tests [58].

The contrasting responses to anxiety-like behaviors observed following chronic and acute administration of AM 630 and JWH 133 confirm the complicated but key role of CB2 receptors in the regulation of anxiety-like behaviors. The distribution of CB2 receptors in brain areas associated with stress and anxiety responses also suggests the involvement of these receptors in the modulation of emotional behaviors. Scientific publications report that additional biochemical studies of the cerebral cortex and amygdala were conducted to determine the mechanisms underlying the above-described behavioral changes. It was proven that the anxiolytic effect induced by chronic administration of AM 630 is associated with increased expression of the gene encoding CB2 in both the cerebral cortex and the amygdala, as well as with reduced expression of the CB2 receptor protein in the cerebral cortex. Interestingly, the anxiolytic effect of JWH 133 was accompanied by opposite changes in CB2 receptors. Chronic administration of JWH 133 decreased expression of the CB2 receptor gene in the amygdala and increased expression of the CB2 receptor protein in the cortex. What is of interest is that, in experiments conducted using transgenic mice with increased expression of CB2 receptors in the CNS, a phenotype resistant to a single exposure to aversive factors used in the LDB and EPM procedures was observed [62,63]. The latest findings confirming the role of CB2 receptors in the regulation of anxiety and mood disorders also include studies showing that mice lacking the gene encoding the CB2 receptor (CB2 −/−) show increased susceptibility to stressful stimuli, assessed in the LBD, EPM and tail suspension test (TST) procedures [62,63,64].

In order to further suggest neurophysiological mechanisms underlying the above-described effects, as we already mentioned, the GABAergic system is considered to be a key element in the regulation of emotional states. Pentameric GABA−A receptors are formed by the assembly of various subunits containing α1, α2, α3 or α5 with β and γ2 subunits. The literature data indicate that GABA−A receptors containing α2 and γ2 subunits mediate the anxiolytic effects of benzodiazepines [65]. Both subunits are located in the limbic system and cerebral cortex [66,67,68]. It has been proven that genetic changes in CB2 receptors also lead to changes in GABA−A receptors. Transgenic mice with increased expression of CB2 receptors also show increased GABA−Aα2 and GABA−Aγ2 gene expression in the hippocampus and amygdala [58]. Scientific reports have indicated the inhibition of GABAergic neurotransmission in the cerebral cortex and hippocampus after the administration of the CB2 receptor agonist JWH 133. These effects can be blocked by prior administration of AM 630. This result supports the thesis of the involvement of CB2 receptors in the effects exerted by JWH 133 on GABAergic transmission and thus the possibility of modulation of GABAergic neurotransmission via CB2 receptors [58].

Summarizing the above-described results, also taking into account the results of our work, it is not possible to clearly determine the influence of CB1 and/or CB2 receptor ligands on anxiety-related behavior because they are dependent on many factors, such as, as mentioned, the dose, the test used or the route of administration. The mechanisms of these phenomena also require explanation, although the indirect influence of CB receptor activation on the activity of other neurotransmitter systems, including GABA and monoaminergic ones, can be taken into account. Further studies are therefore necessary to explain these effects, their type and potential mechanisms. Additionally, a direct modulation of the ECS via ligands of CB receptors could be not effective in treating emotional disorders and is always connected with many adverse effects.

An alternative and promising approach to avoid the adverse effects of direct CB receptor ligands has been focused on evaluating the effects of the indirect activation of the ECS by inhibiting the process of endocannabinoids degradation by the FAAH and MAGL enzymes. Thus, to increase the knowledge in this context, in the second set of our experiments, we evaluated the influence of the indirect modulation of ECS function on anxiety-like responses in mice in the EPM.

In the conducted experiments, after an acute administration of URB 597 (0.3 mg/kg), an FAAH inhibitor, we observed dose-dependent anxiolytic effects in the EPM test in mice. Lower doses may cause a reduction in anxiety while higher doses may produce sedation or other behavioral changes that might confound results. In our studies, we observed an anxiolytic effect of URB 597 only at the dose of 0.3 mg/kg. In turn, after an acute administration of JZL 184 (2, 4, 20 and 40 mg/kg), an MAGL inhibitor, no statistically significant change was observed in the percentage of time spent in the open arms of the EPM, as well as the percentage of the number of entries into these arms.

Our results should be discussed with other available literature data. Some studies suggest that the inhibition of FAAH or/and MAGL could lead to enhanced endocannabinoid signaling and, thus, elevation of AEA or 2-AG could represent potential targets for the development of new classes of pharmacotherapeutics to treat anxiety-related disorders. It is known that both AEA and 2-AG play a key role in the regulation of anxiety-related behaviors, and disruptions in their signaling may lead to increased anxiety [69,70,71]. However, the cited results are not clear and our results of the experiments in the presented work are not quite consistent with the literature data.

It is well established that AEA plays a crucial role in regulating various neurophysiological processes, including responses to stress and anxiety. As one of the main parts of the ECS, AEA has a strong impact on several mechanisms involved in emotional regulation; however, its role in anxiety is complex and context-dependent. This endocannabinoid primarily interacts with CB1 receptors in brain regions such as the hippocampus, prefrontal cortex and amygdala, which can affect the regulation of anxiety-related responses. As we mentioned in the introduction, AEA can produce both calming and anxiogenic effects, depending on the state of the CNS and the presence of other modulating factors. In certain circumstances, elevated AEA levels in the mentioned brain areas may serve as a mechanism for reducing anxiety and promoting emotional balance. However, excessive ECS activity can lead to aversive effects and exacerbate anxiety symptoms, highlighting the complexity of AEA’s role in this process [4,22].

In our experiments, we used URB 597, a selective inhibitor of FAAH, which is responsible for the breakdown of endocannabinoids in the brain, particularly AEA. We can suppose that URB 597, by inhibiting FAAH and increasing AEA levels, activated CB1 receptors, which are mostly implicated in the regulation of fear and anxiety responses, especially those located in brain regions like the hippocampus, amygdala and prefrontal cortex. Our results are consistent with other scientific reports. For instance, the literature data indicate that URB 597 and its analog URB 532 produced anxiolytic-like effects in several animal models, including the LDB and EPM test. These effects were blocked by the administration of CB1 receptor antagonist rimonabant [70]. Moreover, both compounds increased AEA levels in the brain without modifying those of the second endogenous cannabinoid, 2-AG. It is therefore likely that their pharmacological actions, which are sensitive to the CB1 antagonist rimonabant, are primarily due to AEA accumulation. However, other FAAH inhibitors, e.g., ST 4070, also produced an anxiolytic effect after intragastric administration in CD1 mice in the EPM test and LDB test [16,72], and increased the level of AEA and two AEA analogs, N-palmitoylethanolamine oleoylethanolamide, whose biological effects are independent of CB1 receptors [16,70,72,73,74]. Therefore, we should not exclude other mechanisms related to the action of FAAH inhibitors.

Additionally, it has been proven that URB 597 shows exceptional selectivity toward FAAH while showing no affinity toward other ECS elements. Thanks to this, administration of URB 597 selectively inhibits FAAH activity and significantly increases the level of AEA in the brain. Due to this property, it does not cause a number of adverse classic effects accompanying the administration of direct CB receptor ligands, i.e., catalepsy or hypothermia, which are symptoms of cannabinoid poisoning in a rodent [70,75,76,77]. The results of the presented experiments suggest that AEA participates in the modulation of emotional states and indicate the inhibition of FAAH as an innovative approach to anti-anxiety therapy.

However, not only AEA but also another major endocannabinoid, 2-AG, could activate CB1 receptors in the brain, particularly in regions involved in emotional processing, such as the amygdala and prefrontal cortex, and thus can modulate anxiety-related behavior. Unlike AEA, which mainly interacts with CB1 receptors, 2-AG acts on both CB1 and CB2 receptors, suggesting a broader role in modulating brain functions associated with these types of emotions. The literature data indicate that 2-AG can either reduce or increase anxiety symptoms, depending on its levels and the specific neurophysiological conditions [69,71]. Unfortunately, the effects of 2-AG on anxiety have been difficult to ascertain due to its rapid in vivo metabolism. MAGL is the primary enzyme responsible for 85% of 2-AG catabolism, and MAGL inhibitors prevent the breakdown of 2-AG, leading to increased 2-AG levels and potential anxiolytic effects. However, it should be noted that other enzymes, such as ABHD6 and ABHD12, contribute to 2-AG degradation; thus, they have distinct intracellular distributions [71]. As such, it is possible that the inhibition of these minor 2-AG catabolic enzymes may also affect anxiety-related behavior in ways that differ from MAGL inhibition.

In our experiments, we assessed the effect of the inhibition of MAGL on anxiety-related responses in mice observed in the EPM test. We used JZL 184, which is a potent and selective MAGL inhibitor. JZL 184 [29] significantly inhibits MAGL in vivo and is highly selective (300-fold higher for MAGL than for FAAH), with long-lasting effects (>24 h) [29,78].

Unfortunately, in our studies, we did not demonstrate any effect of JZL on anxiety-related behavior in mice. However, there are some literature data describing the anxiolytic potential of MAGL inhibitors, including JZL 184 [78,79,80,81]. Sciolino et al. [81] reported that chronic administration of JZL 184 (8 mg/kg per day for six days) produced anxiolytic-like effects in the EPM under high, but not low, levels of environmental aversiveness. These anxiolytic effects of JZL 184 were prevented by the CB1 inverse agonist rimonabant. The amygdala seems to be critical to this response because the local infusion of an MAGL inhibitor into this structure recapitulates the effects of systemic MAGL blockade [79]. In turn, Ivy et al. [82] revealed that systemic administration of JZL 184 (4, 8 and 16 mg/kg, ip) caused dose-dependent significant anxiolytic-like effects in rats, as demonstrated in the EPM test and marble burying tests, but, surprisingly, the effect of JZL 184 was prevented by co-administration of the CB2 inverse agonist AM630, but not rimonabant. Additionally, many other effects induced by 2-AG, including a reduction in GABA transmission, are independent of mechanisms related to CB1 receptors. It has been revealed that these effects are mainly induced by CB2 receptors, as they are not blocked by the CB1 receptor antagonist LY 320135, but by the CB2 receptor antagonist AM 630 [83].

The contradictory results presented in the experiments discussed above may result from significant differences between the test procedures used, the experimental conditions and the species of animals used in these studies. The anxiolytic effects of JZL 184 may be context-dependent, meaning that they are influenced by environmental factors, such as stress levels or the presence of stressors. Additionally, further investigations are necessary to assess the molecular mechanism of endocannabinoids mobilization and its connection with long-term anxiety responses.

## 4. Materials and Methods

### 4.1. Animals

The experiments were carried out on white naïve male Swiss mice obtained from the Center for Experimental Medicine, Lublin, Poland, weighing from 20 to 25 g. The animals were housed in groups of 10 mice/home cage and maintained under standard laboratory conditions (12 h light/dark cycle, room temperature 21 ± 1 °C) with free access to tap water and laboratory chow (Bacutil, Motycz, Poland). The animals were adapted to laboratory conditions for a period of one week. The number of animals in each group was 8–10 mice. All behavioral experiments were carried out between 8:00 a.m. and 3:00 p.m., maintaining the natural day–night cycle.

The research was carried out with the consent and in accordance with the requirements of the ARRIVE guidelines to improve the reporting of animal research and improve the quality of the studies, and was conducted in accordance with the National Institute of Health Guidelines for the Care and Use of Laboratory Animals, and with the European Community Council Directive for the Care and Use of Laboratory Animals of 22 September 2010 (2010/63/EU). Furthermore, we obtained the agreement of the Local Ethical Committee for all performed experiments: Local Ethical Committee for Animal Experiments in Lublin: Approval Code: 3/2020; Approval Date: 24 February 2020.

### 4.2. Drugs

The compounds acting directly on the ECS were as follows:

- Oleamide (substance, cis-9-octadecenamide, Bio-Techne, Warsaw, Poland): 5; 10; 20 mg/kg—CB1 receptor agonist;
- AM 251 (substance, N-(piperidin-1-yl)-5-(4-iodophenyl)-1-(2,4-dichlorophenyl)-4-methyl-1H-pyrazole-3-carboxamide], Bio-Techne, Warsaw, Poland): 0.25; 0.5; 1; 3 mg/kg—CB1 receptor antagonist;
- WIN 55,212-2 (substance, [R(+)-[2,3-dihydro-5-methyl-3-[(morpholinyl)methyl]pyrrolol [1,2,3-de]-1,4-benzoxazin-yl]-(1-naphthalenyl) methanone mesylate], Bio-Techne, Warsaw, Poland) 0.25; 0.5; 1 mg/kg—CB1/CB2 receptor agonist;
- JWH 133 (substance, 3-(1′1′Dimethylbutyl)-1-deoxy-Δ8-tetrahydrocannabinol, Bio-Techne, Warsaw, Poland): 0.25; 0.5; 1; 2 mg/kg—CB2 receptor agonist;
- AM 630 (substance, (6-iodo-2methyl-1-[2-(morpholinyl)ethyl]-1H-indol-3-yl](4-methoxyphenyl)methanone, Bio-Techne, Warsaw, Poland): 0.25; 0.5; 1;2; 3 mg/kg—CB2 receptor antagonist.

The compounds acting indirectly on the ECS were as follows:

- URB 597 (substance, [3-(3-Carbamoylphenyl)phenyl] N-cyclohexylcarbamate, Bio-Techne, Warsaw, Poland): 0.1; 0.3; 1 mg/kg—FAAH inhibitor;
- JZL 184 (substance, (4-nitrophenyl-4-[bis(1,3-benzodioxol-5-yl)(hydroxy) methyl] piperidine-1-carboxylate, (Bio-Techne, Warsaw, Poland): 2; 4; 8; 20; 40 mg/kg—MAGL inhibitor.

CB receptor ligands and inhibitors of enzymes were suspended in a 1% solution of Tween 80 (Sigma, St. Louis, MO, USA) in a saline solution (0.9% NaCl) and were administered intraperitoneally (ip) at a volume of 10 mL/kg. Fresh drug solutions were prepared on each day of experimentation. Control groups received injections of saline with Tween 80 at the same volume (vehicle) and by the same route of administration.

### 4.3. Experimental Procedure

Mice behavior was observed in the EPM test, widely used in behavioral neuroscience to assess anxiety-like responses in rodents. The procedure was similar to the method used by Lister [84,85]. The apparatus of the EPM is made of dark Plexiglas and is composed of four crossed arms, arranged in a “plus” shape, and a central platform (5 × 5 cm) at the intersection of the arms. Two opposing arms of the maze are open (30 × 5 cm) and the other two arms are enclosed with walls (30 × 5 × 15 cm). The maze is elevated 50 cm above the floor to increase the anxiety level of the mice. Experiments using the EPM test were conducted in a quiet, dark room, and the central square of the maze was illuminated evenly with dim red light.

The experimental procedure included placing each tested mouse in the central platform of the EPM in such a way that the front part of the animal’s body faced the closed arm and the animal had opportunity to explore the four arms for 300 s. Entry into one arm was recorded when an animal placed all four paws past the line dividing the central square from the open arms.

Rodents tend to naturally avoid open arms. Observation of animal behavior included measurement of the following:

- Time spent in the open arms of the maze, as a percentage of the total session duration, and the number of entries into the open arms, as a percentage of the total number of entries into open and closed EPM arms—anxiolytic activity was indicated by increases in time spent in open arms or in the number of open arms entries; anxiogenic effects were characterized by decreases in these measures;
- Measurement of the number of entries into the closed arms and the total number of entries into both types of EPM arms, as an indicator of motor activity of animals in this test [86,87].

### 4.4. Treatment

#### 4.4.1. The Influence of an Acute Administration of Compounds Acting Directly on the ECS on Anxiety-Related Responses in Mice in the EPM Test

CB1 receptor agonist oleamide (5; 10; 20 mg/kg), CB1 receptor antagonist AM 251 (0.25; 0.5; 1; 3 mg/kg), CB1/CB2 receptor agonist WIN 55,212-2 (0.25; 0.5; 1 mg/kg), CB2 receptor agonist JWH 133 (0.5; 1; 2 mg/kg) and CB2 receptor antagonist AM 630 (0.25; 0.5; 1; 2 mg/kg) were administered ip once on the day of the test. Observation of anxiety behaviors was carried out for 30 min after injection. The results were compared to the control group that received the vehicle as an acute injection.

#### 4.4.2. The Influence of an Acute Administration of Compounds Acting Indirectly on the ECS on Anxiety-Related Responses in Mice in the EPM Test

FAAH inhibitor URB 597 (0.1; 0.3; 1 mg/kg) and MAGL inhibitor JZL 184 (2; 4; 20; 40 mg/kg) were administered ip once on the day of the test. Observation of anxiety behaviors was carried out for 30 min after injection. The results were compared to the control group that received the vehicle as an acute injection.

Doses of CB–receptor ligands and inhibitors of enzymes degrading endocannabinoids in the brain used for behavioral experiments and procedures were chosen according to those frequently used in the literature and our previous experiences (Table 8).

### 4.5. Statistics

The data are expressed as the means ± SEM. The statistical analyses were performed using one-way analysis of variance (ANOVA). Post hoc comparison of the means was carried out with Tukey’s test for multiple comparisons, when appropriate. The confidence limit of *p* < 0.05 was considered statistically significant.

## 5. Conclusions

Both indirect and direct pharmacological intervention in ECS functions are able to modulate anxiety-related responses in different ways. Based on our results and available data, it can be suggested that mainly the inhibition of FAAH and MAGL in mice may have a positive influence on the anxiety level, likely due to increased levels of endocannabinoids (AEA and 2-AG), which activate CB1 and/or CB2 receptors and modulate anxiety-related circuits in the brain. Thus, indirect modulation of ECS function (via the inhibition of enzymes degrading endocannabinoids in the brain) could show promise as an anxiolytic target. Following that, more research is needed to fully understand the therapeutic potential and safety profile of possible inhibitors for anxiety-related disorders treatment, as their effects can vary depending on factors such as dosage, chronicity and the context of anxiety-related problems. Our research can contribute to understanding this issue and brings new data that deepen the existing knowledge on this subject.

## Figures and Tables

**Table 1:** Mean (+/− SEM) number of entries into EPM closed arms 30 min after an acute ip injection of oleamide (5–20 mg/kg) or vehicle in mice; *n* = 8.

Acute Drug Injection	Mean Entries into EPM Closed Arms	Number of Mice
vehicle	12.13 ± 1.27	8
oleamide (5.0 mg/kg)	10.25 ± 3.65	8
oleamide (10.0 mg/kg)	8.29 ± 5.94	8
oleamide (20.0 mg/kg)	7.14 ± 4.67	8

**Table 2:** Mean (+/− SEM) number of entries into EPM closed arms 30 min after an acute ip injection of AM 251 (0.25–3 mg/kg) or vehicle in mice; *n* = 8.

Acute Drug Injection	Mean Entries into EPM Closed Arms	Number of Mice
vehicle	12.13 ± 1.27	8
AM 251 (0.25 mg/kg)	8.88 ± 3.0	8
AM 251 (0.5 mg/kg)	9.13 ± 2.53	8
AM 251 (1 mg/kg)	8.5 ± 3.07	8
AM 251 (3 mg/kg)	12.13 ± 3.0	8

**Table 3:** Mean (+/− SEM) number of entries into EPM closed arms 30 min after an acute ip injection of WIN 55,212-2 (0.25–1 mg/kg) or vehicle in mice; *n* = 8.

Acute Drug Injection	Mean Entries into EPM Closed Arms	Number of Mice
vehicle	12.13 ± 1.27	8
WIN 55,212-2 (0.25 mg/kg)	12.50 ± 1.15	8
WIN 55,212-2 (0.5 mg/kg)	12.70 ± 0.94	8
WIN 55,212-2 (1 mg/kg)	9.4 ± 1.07	8

**Table 4:** Mean (+/− SEM) number of entries into EPM closed arms 30 min after an acute ip injection of JWH 133 (0.5–2 mg/kg) or vehicle in mice; *n* = 8–10.

Acute Drug Injection	Mean Entries into EPM Closed Arms	Number of Mice
Vehicle	12.13 ± 1.27	8
JWH 133 (0.5 mg/kg)	10.88 ± 1.13	8
JWH 133 (1 mg/kg)	12.50 ± 1.28	10
JWH 133 (2 mg/kg)	12.80 ± 0.68	10

**Table 5:** Mean (+/− SEM) number of entries into EPM closed arms 30 min after an acute ip injection of AM 630 (0.25–2 mg/kg) or vehicle in mice; *n* = 8–10.

Acute Drug Injection	Mean entries into EPM Closed Arms	Number of Mice
vehicle	12.13 ± 1.27	8
AM 630 (0.25 mg/kg)	12.75 ± 3.49	8
AM 630 (0.5 mg/kg)	10.30 ± 1.56	10
AM 630 (1 mg/kg)	10.70 ± 1.23	10
AM 630 (2 mg/kg)	11.10 ± 1.21	9

**Table 6:** Mean (+/− SEM) number of entries into EPM closed arms 30 min after an acute ip injection of URB 597 (0.1–1 mg/kg) or vehicle in mice; *n* = 8.

Acute Drug Injection	Mean Entries into EPM Closed Arms	Number of Mice
vehicle	12.13 ± 1.27	8
URB 597 (0.1 mg/kg)	10.00 ± 1.60	8
URB 597 (0.3 mg/kg)	10.43 ± 4.24	8
URB 597 (1 mg/kg)	11.29 ± 1.50	8

**Table 7:** Mean (+/− SEM) number of entries into EPM closed arms 30 min after an acute ip injection of JZL 194 (2–40 mg/kg) or vehicle in mice; *n* = 8.

Acute Drug Injection	Mean Entries into EPM Closed Arms	Number of Mice
vehicle	12.13 ± 1.27	8
JZL 184 (2 mg/kg)	10.13 ± 2.53	8
JZL 184 (4 mg/kg)	9.71 ± 2.50	8
JZL 184 (20 mg/kg)	8.43 ± 4.31	8
JZL 184 (40 mg/kg)	8.43 ± 3.51	8

**Table 8:** List of literature data on the basis of which doses and experimental methods were determined.

Compounds	Selected Doses	Literature Data
Oleamide	5; 10; 20 mg/kg	[42,44]
AM 251	0.25; 0.5; 1; 3 mg/kg	[42,43,46,47,51,53]
WIN 55,212-2	0.25; 0.5; 1 mg/kg	[25,26,46]
JWH 133	0.5; 1; 2 mg/kg	[42,43,62,63,64]
AM 630	0.25; 0.5; 1; 2 mg/kg	[42,43,62,63,64]
URB 597	0.1; 0.3; 1 mg/kg	[20,21,69,70,71,88,89]
JZL 184	2; 4; 20; 40 mg/kg	[20,21,78,79,80,81,88,89]

## Data Availability

Data are contained within the article.
